# Age-Related Variation in Cardiovascular Risk Associated With Red Cell Distribution Width

**DOI:** 10.1093/geroni/igaa057.1688

**Published:** 2020-12-16

**Authors:** Jarrod Dalton, David Zidar, Sadeer Al-Kindi, Nikolas I Krieger, Adam Perzynski

**Affiliations:** 1 Cleveland Clinic Lerner College of Medicine at Case Western Reserve University, Cleveland, Ohio, United States; 2 Louis Stokes Cleveland VA Medical Center, Cleveland, Ohio, United States; 3 University Hospitals Cleveland Medical Center, Cleveland, Ohio, United States; 4 Lerner Research Institute - Cleveland Clinic, Cleveland, Ohio, United States; 5 Case Western Reserve University at MetroHealth, Cleveland, Ohio, United States

## Abstract

We previously showed that routine indicators of immune-hematologic function are strongly associated with all-cause and cardiovascular mortality, especially among older adults, in whom traditional atherosclerotic cardiovascular disease (ASCVD) risk factors predict poorly. The objective of this study was to quantify differences in the relationship between red cell distribution width (RDW) and 5-year risk of ASCVD events (stroke, myocardial infarction or cardiovascular death) as a function of age and the area deprivation index (ADI). We analyzed electronic health records of 76287 Cuyahoga County, Ohio residents who were over age 40 and who visited Cleveland Clinic Health System and/or MetroHealth System in two consecutive years between 2005 and 2015, the latter of which served as the index/baseline. Multivariable Cox regression was used, adjusting for sex, race/ethnicity, diabetes, systolic blood pressure and antihypertensive use. Generally, RDW levels in disadvantaged neighborhoods corresponded to people 15-20 years older from affluent neighborhoods. In a main-effects-only model, we found higher ASCVD event rates associated with age (hazard ratio [95% confidence interval] per 10 year increment: 1.34 [1.32–1.35]), ADI (top vs. bottom quintile: 1.30 [1.24–1.36]) and RDW (>16% vs ≤13%: 2.03 [1.94–2.12]). Age and RDW exhibited a synergistic interaction (χ2 = 17.7 on 2 df, p<0.001), with slightly larger RDW effects associated with increasing age, while evidence of differential RDW effects across ADI quintiles was weak (χ2 = 12.9 on 8 df, p=0.11). We conclude that RDW effects on ASCVD event risk are large, independent from traditional ASCVD risk factors and increase with advancing age.

